# Constructing Wholeness in LGBTQ+ Healthcare Access: A Grounded Theory Model

**DOI:** 10.3390/healthcare14040536

**Published:** 2026-02-22

**Authors:** Braveheart Gillani, Jessamyn Moxie, Meagan Ray-Novak, Roni Diamant-Wilson, Dana M. Prince, Laura J. Mintz, Scott Emory Moore

**Affiliations:** 1 College of Health and Human Services, School of Social Work, Violence Prevention Center, University of North Carolina, Charlotte, NC 28223, USA; braveheart@charlotte.edu; 2 College of Health and Human Services, Department of Epidemiology and Community Health, Violence Prevention Center, University of North Carolina, Charlotte, NC 28223, USA; 3 Social Work Department, Westfield State University, Westfield, MA 01086, USA; mraynovak@westfield.ma.edu; 4 Jack, Joseph & Morton Mandel School of Applied Social Sciences, Case Western Reserve University, 10900 Euclid Ave, Cleveland, OH 44106, USA; 5 Internal Medicine-Pediatrics, Case Western Reserve University, 9501 Euclid Ave, Suite #0111, Cleveland, OH 44106, USA; 6 Frances Payne Bolton School of Nursing, Case Western Reserve University, Cleveland, OH 44106, USA

**Keywords:** LGBTQ+, grounded theory, qualitative research, healthcare access, wholeness, intersectionality, minority stress theory, gender-affirming care, intra-community support, self-advocacy

## Abstract

**Highlights:**

**What are the main findings?**

- LGBTQ+ individuals’ engagement with healthcare across the life course is shaped by a dynamic process of reconstructing and deconstructing forces, wherein affirming care, accessible providers, and community support promote wholeness, while discrimination, structural barriers, and dismissal fragment identity and well-being.
- Despite diverse identities and life stages, participants described a shared, non-linear process of moving toward wholeness through four interconnected internal mechanisms: interconnecting selves (identity integration and authenticity), intra-community support, self-determined care (advocacy, persistence, and pursuit of quality care), and meaning-finding.

**What are the implications of the main findings?**

- Healthcare systems and providers must move beyond deficit-based or purely biomedical approaches and instead adopt wholeness-oriented, affirming models of care that recognize the interconnected social, emotional, cultural, and identity-based dimensions shaping LGBTQ+ health experiences.
- Strengthening LGBTQ+ community infrastructures and peer-based knowledge exchange is critical, as intra-community support functions as a key mechanism for navigating healthcare, mitigating harm, and fostering resilience—particularly in contexts where formal healthcare systems remain inequitable or unresponsive.

**Abstract:**

LGBTQ+ individuals continue to experience substantial barriers to accessing affirming healthcare, including discrimination, structural inequities, and provider-level limitations. This study aimed to develop an emergent grounded theory model of constructing wholeness in healthcare. Methods: This study employed a secondary constructivist grounded theory analysis of qualitative data from The Rainbow Connections Study, a community-based system dynamics project. Data were collected through eight group model-building sessions conducted via Zoom with 28 LGBTQ+ participants, including older adults, youth, transgender and gender-diverse individuals, and staff from the LGBTQ+ community center who also held service and practitioner roles; analytic claims are framed to reflect this mixed-role sample. Sessions were audio- and video-recorded, transcribed verbatim, and analyzed using open, axial, and selective coding procedures. Constant comparative methods, reflexive memoing, and member checking were used to support analytic rigor and trustworthiness. Results: Analysis revealed a dynamic process in which LGBTQ+ individuals encounter external forces within healthcare systems that alternately support or fragment their sense of self. In response, participants engaged in four interconnected internal processes—interconnecting selves, intra-community support, self-determined care, and meaning-finding—that facilitated movement toward wholeness. These processes were non-linear, iterative, and present across diverse identities and life stages. Conclusions: The emergent theory of Constructing Wholeness in Connecting to Healthcare highlights that LGBTQ+ healthcare experiences extend beyond access and utilization to include identity integration, community reliance, and meaning making. Supporting LGBTQ+ health requires healthcare approaches that affirm wholeness, reduce structural harm, and recognize the central role of community in navigating care.

## 1. Introduction

Lesbian, gay, bisexual, transgender, queer, questioning, and other people, including pansexual and two-spirit people (LGBTQ+), are disproportionately affected by adverse health outcomes [1,2,3,4,5,6,7,8,9,10]. LGBTQ+ identified individuals report discrimination, harmful interactions with healthcare providers, and difficulty accessing appropriate care [11,12]. LGBTQ+ communities rely heavily on one another for support, guidance, and access to affirming healthcare [13,14]. While prior research has documented multiple facilitators and barriers, less is known about the mechanisms through which these factors combine over time to shape sustained connection or disengagement from care. Qualitative work has shown how LGBTQ+ subgroups (e.g., youth/young adults, elders, and BIPOC individuals) navigate healthcare and resist discrimination in medical settings [15,16,17,18,19]. However, limited studies have examined how diverse members of the LGBTQ+ community engage with healthcare across the life course.

The life course perspective seeks to account for the interconnectedness of life domains (such as socio-political, global, and social events/eras; family; education; work; and health) and how they influence one another in an individual’s life [20]. Wholeness is an important, crucial, and essential concept in the life course perspective, which emphasizes the interconnectedness of an individual’s life aspects and the need to consider the whole person rather than just individual parts [21]. In this study, we use data from a larger pilot, The Rainbow Connections Study, which explored connections and disconnections from care for LGBTQ+ groups across their life course, utilizing system science methods. It applied Community-Based System Dynamics (CBSD) to identify factors that connect and disconnect LGBTQ+ communities to physical and mental healthcare [22]. The pilot study aimed to understand the barriers and facilitators experienced by LGBTQ+ populations in accessing physical and mental healthcare from a complex systems science perspective. In this secondary analysis, we applied a grounded theory approach to develop an emerging theory of how LGBTQ+ individuals achieve wholeness within themselves and their communities. The grounded theory led to the development of a “wholeness” model that identifies a nonlinear process by which LGBTQ+ individuals move towards wholeness within themselves. Our grounded theory model specifies how internal mechanisms (e.g., identity integration, intra-community support, self-determined care, and meaning finding) operate in response to reconstructing and deconstructing healthcare forces.

## 2. Background and Theoretical Framework

Social groups profoundly impact the development of individual identity [23]. The importance of group identity, connection with other LGBTQ+ individuals, and the larger affiliative group cannot be understated. Group identity is complex and often entwined with social identities such as race, ethnicity, age, (different) ability, and the development of sexuality and gender identity [24]. Positive relational factors have been shown to offset the adverse effects of societal disadvantage for LGBTQ+ individuals [25,26,27].

### 2.1. Connection with Healthcare

Facilitators of healthcare connection vary across LGBTQ+ subpopulations, indicating their unique intersectional positionality within specific societies and societal understandings. LGBTQ+ young adults have been found to prioritize collective action and efforts to affect social change to combat the effects of discrimination and facilitate hope [15,16]. Having spaces where youth feel accepted and connected with other LGBTQ+-identified youth is restorative, allowing youth a “recharging station” [15]. Developmentally, LGBTQ+ young adults desire to assert themselves in decision-making, identity, and self-efficacy. The ability to choose healthcare providers and pathways of care, and to make independent decisions, allows them to feel in control of their lives [15,17]. Having safe, stable adults with whom LGBTQ+ young adults can engage in community-making provides opportunities to feel “seen” and understood, a critically important factor considering the increased rupture in relationships with the family of origin for LGBTQ+ young adults [15,16].

Over the past decade, increased inclusion of LGBTQ+ healthcare into mainstream healthcare systems has helped older LGBTQ+ adults connect with healthcare [28]. For example, older lesbians report that inclusive signage and representation of LGBTQ+ community members within the healthcare system support their connection to healthcare [29]. Connection with empathetic, LGBTQ+-affirming healthcare and social services providers improves the quality of life among LGBTQ+ older adults [30].

For transgender and gender-diverse (TGGD) individuals, the availability of, and access to, trans-specific healthcare is critical for receiving relevant gender-affirming medical and mental healthcare [31,32,33,34]. The need for gender-affirming healthcare spans the life course. Among older TGGD adults, the intersection of cisgenderism, homophobia, and ageism impacts healthcare engagement [35,36]. Explicit acknowledgment of prior trauma and assurance of open communication increase TGGD individuals’ engagement in healthcare [37]. Other facilitators include inclusion and visibility of TGGD individuals and the creation of safe and affirming healthcare environments [38,39].

Collectively, these facilitators indicate that connection with healthcare depends on more than isolated, affirming encounters. Rather, connection is shaped by how healthcare experiences interact with identity integration, community support, self-determination, and meaning across the life course. This suggests the need for a process-oriented understanding of how LGBTQ+ individuals move toward connection and coherence within healthcare systems.

Across studies, cisgender LGB participants more often emphasize interpersonal affirmation and disclosure safety. In contrast, transgender, nonbinary, and gender-diverse participants highlight the importance of gender recognition and provider competence in shaping connection. Taken together, this literature demonstrates that connection with healthcare is supported by more than structural access alone. Prior studies emphasize the importance of affirming interactions, trust, community-derived knowledge, and alignment between healthcare encounters and individuals’ sense of self. These findings provide an empirical foundation for understanding how connections may be actively constructed over time.

### 2.2. Disconnection with Healthcare

Different subpopulations within the LGBTQ+ community also experience distinct barriers to healthcare. LGBTQ+ youth describe societal stigma, homophobia, and transphobia as deterrents to healthcare engagement [40]. Barriers to healthcare engagement likewise differ across LGBTQ+ subpopulations, reflecting distinct forms of vulnerability and exposure to harm. A lack of empathy and understanding from healthcare providers further impedes connection [41,42].

For older LGBTQ+ adults, societal change, discriminatory policies, and historical oppression are particularly salient [43,44]. Physical health complications, social isolation, and exclusion from families of origin compound barriers to care [45,46]. Additional barriers include fear of mistreatment [47,48,49], financial constraints [50,51,52], and transportation challenges [53,54,55,56].

TGGD individuals experience both individual and structural discrimination in healthcare settings [41,57,58]. Barriers include denial of care, lack of provider training, constrained provider choice, pathologizing requirements for gender-affirming care, and emotional fatigue from educating providers [57,58,59,60]. Gender-diverse and non-binary individuals encounter additional barriers, including binary assumptions by providers, inadequate reproductive healthcare support, and limited preventive care standards [61,62].

LGBTQ+ individuals with intersectional identities experience compounded marginalization. Black transwomen experience disproportionately high rates of HIV and STIs [63], dramatically reduced life expectancy [64], and intersecting effects of racism, misogyny, and transphobia. Black and Latina lesbians experience worse birth outcomes [65], and racial minority older adults experience poorer health outcomes overall [66].

Taken together, these barriers illustrate how healthcare systems can function as deconstructing forces that fragment identity, undermine agency, and disrupt continuity of care. When healthcare encounters threaten rather than affirm core aspects of self, individuals are pushed away from engagement and toward protective withdrawal. This contrast between reconstructing and deconstructing experiences underscores the need for a model that explains how LGBTQ+ individuals navigate these opposing forces across time.

Conversely, the barriers described above show how healthcare can become a site of fragmentation that pushes individuals away from engagement, even when care is clinically indicated or strongly desired. These barriers often compound rather than occur in isolation: structural constraints (e.g., access, cost, availability) intersect with interpersonal harms (e.g., dismissal, misgendering, assumptions, stigma) to erode trust, increase anticipated harm, and intensify the perceived risks of disclosure. Over time, repeated negative encounters can accumulate into protective strategies, delayed care, selective disclosure, or disengagement—that reduce exposure to harm but also undermine continuity and preventive care. While cisgender LGB individuals frequently report anticipated stigma and dismissal, transgender, nonbinary, and gender-diverse individuals disproportionately encounter barriers related to misgendering, gatekeeping, and lack of provider knowledge [67]. Taken together, the literature suggests that disconnection emerges through cumulative “deconstructing” experiences that destabilize identity safety and epistemic trust [68,69] within healthcare, highlighting the need for a process-oriented framework that explains how individuals move between connection and disconnection over time and under what conditions reconnection becomes possible.

Across both facilitators and barriers, interpersonal exchanges with healthcare providers emerge as a central mechanism shaping connection and disconnection with care. Provider attitudes, clinical knowledge, and cultural competence can either exacerbate or mitigate structural and interpersonal barriers, influencing whether healthcare encounters are experienced as affirming or harmful [70]. Negative provider characteristics—such as dismissal of patient concerns, misgendering, reliance on heteronormative or cisnormative assumptions, and lack of familiarity with LGBTQ+ health needs—may intensify anticipated stigma and reinforce disengagement, even when services are formally available. Conversely, affirming provider behaviors, including respectful communication, willingness to learn from patients’ lived expertise, and provision of knowledgeable, gender-affirming care, have the potential to ameliorate barriers and foster trust and continuity [71].

Prior qualitative studies have richly documented the lived experiences of LGBTQ+ individuals navigating health services, highlighting how stigma, disclosure risk, provider assumptions, and structural constraints shape healthcare engagement [72]. This body of work illustrates how individuals negotiate safety and affirmation in clinical encounters, rely on informal networks to identify affirming providers, and adapt their healthcare-seeking behaviors in response to prior negative experiences. Qualitative research has also underscored substantial heterogeneity across LGBTQ+ subpopulations, with transgender, nonbinary, and gender-diverse individuals often encountering distinct challenges related to gender recognition, gatekeeping, and provider knowledge [72,73]. While these studies provide critical insight into barriers and facilitators of care, they have tended to focus on discrete experiences rather than theorizing how individuals move over time between connection, disconnection, and re-engagement with healthcare. The present study builds on this qualitative literature by offering a process-oriented grounded theory that situates these lived experiences within a dynamic model of constructing wholeness.

### 2.3. Theoretical Frameworks

While the above literature identifies key facilitators and barriers to LGBTQ+ healthcare engagement, it does not fully explain how these elements interact over time to shape sustained connection, disengagement, or re-engagement with care. To interpret these empirical patterns and guide theory development, we draw on three complementary theoretical frameworks: Minority Stress Theory, the Wellness Wheel, and intersectionality.

This study draws on Minority Stress Theory (MST), the Wellness Wheel/Indivisible Self Model of Wellness, and intersectionality as grounding frameworks [6,8,9,74,75,76,77]. MST explains heightened adverse health outcomes among LGBTQ+ populations due to chronic exposure to discrimination and stigma across social levels [8,9,78,79]. Empirical research demonstrates strong links between minority stress and adverse mental health outcomes [8,9,25,74].

The Wellness Wheel emphasizes wholeness as central to optimal well-being and conceptualizes wellness as an integration of identity, meaning, and life roles [75,80,81,82,83,84]. Intersectionality provides a lens for understanding how multiple marginalized identities shape healthcare access and power relations [76,84].

Wholeness has been conceptualized as integration of self and meaning across life domains [75,80,81,82,85,86,87,88,89]. Fragmentation arises from repeated discrimination and invalidation, undermining self-trust and system navigation [86,87], whereas integration supports agency, advocacy, and resilience [85,88,89,90,91]. These processes are particularly salient for LGBTQ+ individuals navigating healthcare systems amid structural inequities.

We used these three frameworks together because no single theory adequately captures the complexity of LGBTQ+ healthcare engagement across the life course. Minority Stress Theory explains stress-related pathways, the Wellness Wheel conceptualizes wholeness as integration across life domains, and intersectionality reveals how power operates across interlocking systems of oppression to shape lived experience, access to resources, and differential health and wellbeing outcomes.

### 2.4. Study Aim and Research Questions

To understand LGBTQ+ healthcare disparities, it is critical to examine how barriers and facilitators interconnect across the life course [28]. This study aims to explore how diverse LGBTQ+ individuals experience connection and disconnection with healthcare and to develop a grounded theory model of how they construct wholeness in this process.

The research questions were:

- What is it like for LGBTQ+ individuals and stakeholders to connect with healthcare systems across the life course?
- What is it like for LGBTQ+ individuals and stakeholders to disconnect from healthcare systems across the life course?

This secondary grounded theory analysis yielded a theory of constructing wholeness that extends beyond connection and disconnection alone.

## 3. Methods

### 3.1. Sample and Recruitment

This manuscript is part of a multi-phase project that included experts, scholars, and community members. Participants in the parent study were 18 years of age or older. They were purposively recruited because of their direct relationships with the subject matter at hand through (a) being an interested party (i.e., a potential LGBTQ+-identifying individual connected to the LGBTQ+ Center of a small midwestern city in the US), (b) a practitioner serving the LGBTQ+ community, or (c) being a medical, behavioral health, or dental provider serving the LGBTQ+ community.

The parent study (Rainbow Connections) included a series of group model-building sessions to explore connections and disconnections experienced by LGBTQ+ individuals across their life course. Group model building is a participatory methodology designed to engage communities in developing visual models through rich storytelling, in-time investigation of emergent factors, and the transmission of systems thinking [22]. IRB approval was obtained from the researchers’ university for the study (STUDY20200359). Prior to data collection, all participants read the consent forms and provided verbal confirmation. No written consent forms were obtained, as the study was conducted via Zoom due to COVID-19 pandemic restrictions. National Institutes of Health, National Center for Advancing Translational Sciences, Clinical and Translational Science Award grant, UL1TR002548, provided funding for this study. The funders did not play any role in data collection, interpretation, or presentation.

Participants in the parent study were recruited through community partnerships and existing community support groups at an LGBTQ+ Center. Project team members developed flyers and, with permission, attended community support group sessions to recruit attendees for the project. Data-gathering sessions were organized either at the same time as the regular group meeting or at a different time decided by the group. For this analysis, participants were categorized by primary role at the time of participation: (a) LGBTQ+ service users, (b) LGBTQ+ community staff, and practitioners serving LGBTQ+ communities. Sexual orientation and gender identity were self-reported during group sessions and confirmed during transcription review. Further details of the sample are provided in Table 1.

### 3.2. Data Collection

Original data collection for the Rainbow Connections Study was completed with four groups associated with the LGBT Center of a small Midwestern city: older LGBTQ+ adults, LGBTQ+ youth, transgender and gender-diverse (TGGD) individuals, and staff of the LGBTQ+ Center who also belonged to the LGBTQ+ community. Each group participated in two data collection sessions. Sessions included structured and unstructured activities facilitated by trained researchers, including guided prompts, turn-taking support, and collective reflection. The first session focused on identifying primary facilitators and barriers to healthcare engagement. Sessions lasted approximately 1.5–2 h and were audio- and video-recorded via Zoom.

The facilitation team (authors 1, 2, and 6) provided a summary of findings from session one during session two and invited participant feedback, corrections, and additional nuance. Sessions included both structured and unstructured activities to elicit in-depth narratives and experiential accounts of participants’ interactions with healthcare systems.

Data for the present manuscript were derived from a secondary analysis of these session transcripts. Due to the richness and depth of participant narratives, the research team conducted post hoc transcription, coding, and grounded theory analysis beyond the original scope of the CBSD project. All participants were invited via email to participate in a member-checking focus group. Although only four participants attended, each represented one of the original four groups, enabling meaningful cross-group validation of the emerging theory.

### 3.3. Analysis

This study employed a constructivist grounded theory approach, informed by but not fully aligned with Straussian procedural coding, to support theory generation from a secondary qualitative corpus [22,92,93,94,95,96,97,98,99,100,101]. Interpretive grounded theory provided a structured analytic process, including open, axial, and selective coding, to support theory development [93,94]. Constructivist grounded theory served as the primary analytic framework, emphasizing reflexivity, co-construction of meaning, and sociocultural context [99]. Analysis progressed from in vivo and process codes to focused categories, which were iteratively refined through constant comparison and memo-writing. Given the secondary nature of this analysis, constructivist principles guided the re-engagement of transcripts, attending to meanings shaped by both the original CBSD context and analytic interpretation [92,101,102].

All group model-building sessions were transcribed verbatim and independently coded using ATLAS.ti (version 22). An initial codebook was developed, and weekly analytic meetings were held to compare and refine codes. Coding was conducted independently by three analysts, with discrepancies resolved through consensus discussion and return to original excerpts. Open codes were merged, revised, or eliminated as appropriate. Axial coding employed constant comparative methods to develop categories, subcategories, constructs, and themes [92,100]. Process coding, in vivo coding, and descriptive coding were the primary strategies used [96,97]

Reflexive memoing was used throughout the analysis.

Coding initially focused on LGBTQ+ Center staff and youth transcripts. During analysis of older adult transcripts, a shift in language use emerged, with participants emphasizing “connecting to healthcare” rather than “connection to healthcare.” This gerund-based shift informed new developmental and historical codes, capturing change over time and action-oriented meaning [92]. Earlier transcripts were reanalyzed for consistency, consistent with constructivist grounded theory’s iterative approach. Theoretical saturation was determined when no new properties of existing categories emerged across transcripts, rather than through prospective sampling [92,100]. Given the fixed dataset, saturation was assessed retrospectively by repeatedly confirming category boundaries rather than through iterative data collection.

**Figure 1:**
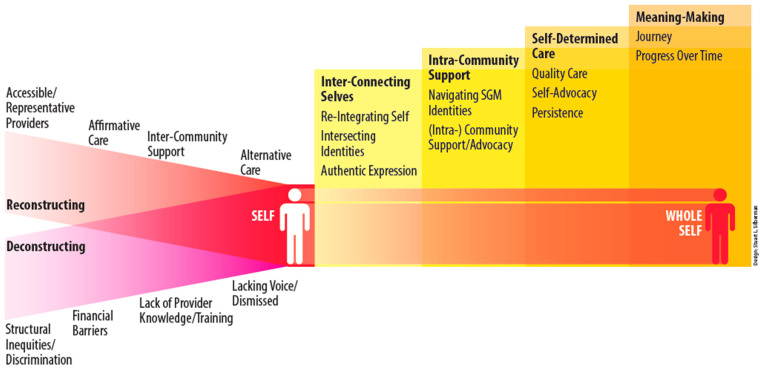
Constructing Wholeness in Connecting to Health Care.

Selective coding examined relationships among four axial categories and identified a core category—Meaning Finding—that theoretically explained participants’ processes of connecting to healthcare. This trajectory is illustrated in Figure 1. Analytic memos were central to identifying the core category and its relationships. The resulting model reflects a co-constructed interpretation rather than an objective truth.

Lincoln and Guba’s criteria for trustworthiness—credibility, confirmability, dependability, and transferability—guided analytic rigor [98,99]. Credibility was enhanced through data triangulation and a member-checking focus group, during which all constructs were reviewed and affirmed by participants [99]. Confirmability was strengthened through reflexive dialogue among lead authors and consensus-based resolution of coding disagreements [99]. Negative case analysis further refined the emerging theory [98].

Dependability was supported through audit trails, detailed field notes, and systematic documentation of analytic decisions: thick description, constant comparison, and saturation enhanced reliability. For transferability, detailed methodological documentation and contextual description were maintained to support replication in similar settings. Transparency regarding research team positionality was emphasized to enhance verisimilitude [98,100]. The team represented diverse identities, life experiences, and social locations, all of which informed recruitment, data collection, analysis, and interpretation.

The team attended to potential analytic bias through reflexive, collaborative, and documented analytic practices. Team members’ positionalities were recognized as shaping analytic sensitivity to experiences of stigma, affirmation, and healthcare engagement. Reflexive memoing was used throughout coding to surface assumptions, and regular analytic debriefs were held to examine divergent interpretations. Disagreements were resolved through return to the data and constant comparison, with all analytic decisions, codebook revisions, and theoretical memos documented in an audit trail to enhance confirmability.

## 4. Results

The grounded theory emerging from this analysis describes how LGBTQ+ individuals navigate and interpret their healthcare experiences across the life course. Participants’ narratives, reflecting both lived experience and embedded community-system perspectives, revealed that reconstructing and deconstructing forces represent external conditions. In contrast, interconnecting selves, intra-community support, self-determined care, and meaning-finding are internal processes activated in response to those conditions. Participants’ narratives revealed two overarching external processes, reconstructing and deconstructing, which alternately support or fragment their sense of self as they encounter medical, institutional, and interpersonal forces. In response to these external conditions, participants engaged in four interconnected internal processes: interconnecting selves, intra-community support, self-determined care, and meaning-finding that enabled them to move toward greater wholeness. These processes were non-linear, iterative, and mutually reinforcing rather than sequential steps. The sections that follow describe the reconstructing and deconstructing forces that shape participants’ healthcare experiences and then elaborate on the internal mechanisms through which they cultivated wholeness despite structural, social, and personal challenges.

Primary findings from this analysis are visualized in Figure 1. The Theory of Constructing Wholeness in Connecting to Healthcare outlines a process by which LGBTQ+ individuals advance their own wholeness while navigating the helpful and harmful structures embedded in healthcare.

LGBTQ+ individuals undergo a series of “reconstructing” and “deconstructing” events in relation to wholeness. The processes of reconstruction and deconstruction result from external events. The reconstructing events include engagement with accessible and representative providers, affirmative care, inter-community support, and alternative care. The deconstructing events include exposure to structural inequalities and discrimination, financial barriers, lack of provider knowledge and training, and a lack of voice or being dismissed. Both the reconstruction and deconstruction themes, along with their sub-elements, were explicitly mentioned across all groups, indicating their ubiquitous presence throughout the life course of LGBTQ+ individuals. In addition to reconstruction and deconstruction, LGBTQ+ individuals described achieving wholeness through a series of individually focused processes. To continue their journey from fragmentation to wholeness, LGBTQ+ individuals navigate a series of themes, none of which is more or less critical or built sequentially on the others. These themes are interconnected selves, intra-community support, self-determined care, and meaning-finding. This manuscript briefly describes the themes of reconstruction and deconstruction, along with their components. It then focuses on the qualitative analysis of how the participants expressed their journey towards wholeness. Further quotes providing each of the reconstruction and deconstruction subthemes are provided in Table 2.

### Reconstructing

Elements within the “reconstructing” and “deconstructing” themes on the model’s left side overlap and iterate, influencing different individuals with varying impacts and potency. Within the reconstructing phase, there is no sharp boundary between accessible and representative providers, affirmative care, inter-community support, or alternative care. The reconstructing arc describes the elements external to oneself that assist in assembling oneself. Research indicates that LGBTQ+ providers often provide affirmative care and develop community-based strategies to support holistic patient care [33,36,37,38,67,70,71]. Affirmative care, inclusive of provider engagement, language, and environment, has been validated as a profound health intervention for LGBTQ+ individuals across their life course [30].

Inter-community support within this model refers to the emotional, social, financial, and physical support extended by other individuals (of varying identities) within the community to members of the LGBTQ+ community. Research indicates that such support has profoundly positive impacts on LGBTQ+ individuals [29]. Finally, alternative care refers to methods outside the traditional medical model that participants use to support their health journeys. These included seeking support from Eastern medicine and accessing hormones and surgery from sources considered “black market.”

*Deconstructing*

The deconstruction process experienced by LGBTQ+ individuals across their life course refers to components external to oneself that fragment or hinder understanding oneself. The deconstruction process was analogous to the reconstructing process, with no sharp boundaries between financial barriers, lack of provider knowledge and training, and lack of voice or being dismissed. Participants shared repeated instances of exposure to structural inequalities and discrimination, leading to experiences of physical and mental health disparities, employment prevention, financial insecurity, and overall lower quality of life [50].

Participants shared research-validated knowledge that LGBTQ+ individuals experiencing financial hardships were often exposed to providers with low amounts of knowledge and training regarding LGBTQ+ experiences and were very often dismissed or disrespected [67]. These financial barriers are often compounded across dimensions of race and gender, with transwomen of color experiencing greater severity of these barriers [64]. Participants further mentioned how race and gender played critical roles related to respect from providers, with racial minorities and those aligning with femininities experiencing further disrespect and dismissal of voice from providers.

*Wholeness in Connecting to Healthcare*

As LGBTQ+ individuals navigate the reconstructing and deconstructing aspects of the medical model, they continue their journey towards developing wholeness. The right side of Figure 1 explains aspects of wholeness, as articulated by the participants and identified through analysis. We identified four main themes and ten subcategories across the eight data collection sessions. The four main themes are inter-connecting selves, intra-community support, self-determined care, and meaning-finding. Each theme, with its constituent constructs, is provided in Table 3. These processes, factors, and all major themes emerged from all the group sessions. Quotations support our findings, and participant-selected pseudonyms protect participants’ identities.

*Interconnecting Selves*

Participants expressed that to express their whole self, participants’ intersecting parts (selves) or identities must be recognized. This theme emerged across all groups, reflecting how participants experienced both support (from the community) and tension (from society and the medical system) in developing their complex identities. This construct had three categories: Integrating (or Re-integrating) Self, Intersecting Identities, and Authentic Expression.

*Integrating (or Re-integrating) Self*

Participants spoke of the importance of integrating (or re-integrating) components of self after being discriminated against by society. Aligning with sexual and gender identities emerged as a critical part of the process of integrating or re-integrating the self. Participants shared both the integration (or reintegration) process and its benefits. This is exemplified when Brandy, a TGGD participant, said:

*“But the assumption is that it’s [alignment with gender identity] not important. It’s not needed. It’s just completely voluntary, and not only was it important for me to align my appearance with how I see myself. But honestly, having that surgery is important for me for a lot of reasons. It improves my survivability because I’m not getting clocked [recognized as trans] everywhere I go.”*

Here, Brandy clearly states that there are multiple compelling reasons for them to seek gender-affirming care. The integration or reintegration they experienced provided them with safety by not getting outed as transgender everywhere they went, but also supported them in self-image and self-perception.

Participants described integration as a process where they identified their self-worth and self-esteem and began to discover their “true selves”. Construction metaphors were consistently used, such as “building” and “brick by brick,” to convey the labor intensity and intricacy of this process. Boris, a member of the elder group, reiterated the same point when he said, *“If you’re reconnecting with yourself, you’re building your self-esteem. You’re rediscovering who you are.”*

*Intersecting Identities*

This construct refers to how participants described their interconnected, marginalized identities. These identities played a salient role in navigating the healthcare system around them while engaging in self-integration practices towards wholeness. Participants reported that navigating different parts of their identities and being marginalized were recurring experiences in their lives. Participants experienced incidents when healthcare providers did not appreciate the complexity of navigating several marginalized identities, potentially increasing the burden of explanation on the patients. This is exemplified when Rose, a member of the Center staff group, said:

“I think about immigrant identity and undocumented people. I have family members who are undocumented. They have never gone to a doctor because of the fear alone. How invisible are they? Right? If this is the level of oppression that we face as queer people, and then as an additional layer, BIPOC queer people. Do you imagine the level of shit…that there is and just accessing something basic that should be available to anybody as a human, for folks that are refugees or immigrants or you know whatever they are, because of these, this f**** up immigration system that we live in.”

Here, the participant identifies several layers of intersectionality: being an immigrant, being BIPOC, being queer, and feeling invisible within the healthcare system. Experiences due to marginalized intersectional identities create feelings of isolation and fear and are exacerbated by racism, xenophobia, and queerphobia.

*Authentic Expression*

Participants prioritized authenticity when navigating the healthcare system and believed they needed to express their “real” selves to get appropriate support (often referring to gender and other identities). Participants described critical interactions with providers where they felt affirmed without having to disclose or justify their expression. Sophia, an LGBTQ+ youth, stated a positive experience within the healthcare system by saying: “I see a family friend who’s a nurse practitioner. I really love her because I don’t have to explain myself. It’s such a unique thing because I don’t feel like I have to seek validation from another person.” Here, Sophia expresses great relief at being able to disclose their identity to their provider without needing to defend or seek validation to obtain medical care.

The authenticity of the individual and self-expression provided the participants with a more profound sense of confidence and trust to navigate the world and connect meaningfully with others. This was expressed by Jasmine, a member of the TGGD group, when she said:

*“We are living our authentic best lives. Something about us glows. Something about us shines. Something about us gets respect. Because we are living our best life. People see this. They understand it. They detect it. The knee-jerk response of any human being that receives love with open hands is to respond with love. I don’t care how hardened you are. Something in your heart [softens].”*

Here, the participant indicates the importance of expressing their authentic identity. They identify the power and beauty of living unabashedly in their true identity, and they firmly believe that people experience connection and empathy as they witness authenticity and joyous lives. Authenticity has greatly improved their quality of life and added a sense of attractiveness to their being, which is recognized and well-received by most others in society. They indicated the impact of authenticity within their physical embodiment (glow) as well as in others (open hands).

*Intra-community Support*

Participants described navigating their LGBTQ+ identities and building intra-community support through information-sharing, advocacy, and education, and personal stories/experiences, aligning with extant research [16,27,103,104]. Participants experienced intra-community support as critical for connecting to healthcare, with two main subcategories: navigating their identities and community support/advocacy.

*Navigating Identities*

Participants shared personal experiences and stories that reflected their within-group/LGBTQ+ identities. Due to diversity and differing amounts of and access to privileges within the broader LGBTQ+ community, navigating identities was critical for the participants’ wholeness. Hence, the community was a space for development and often self-discovery of identity. Within-group community allowed participants to express their identities freely and connect with other LGBTQ+ individuals.

For older adults, these moments of connection were precious and tenuous, often the only opportunity they had to express parts of their identity and meet others with shared experiences in the larger society. This was expressed by Mark, an older adult, when he said:

*“…I never knew I was gay. I came back to [city], and I started going to the gay bars and clubs. Within a year, I met my [partner]. And we’ve been together now 58 years.”*

Mark articulates that he discovered his sexuality with the support of the community within gay bars and clubs, an often-cited component of navigating identities within the LGBTQ+ community. He found his partner within these spaces, allowing him to develop a long-term relationship. Research indicates that gay bars and clubs are “important venues for the formation of social networks and for the development of community among gay men” [105,106]. These spaces have historically been important refuges for LGBTQ+ community members.

*Community Support/Advocacy*

Community support and advocacy emerged as participants expressed navigating systemic oppression and stigma through connection with community safe spaces and collective support. Community support and advocacy were emphasized by the participants, potentially because the groups were held within a community support space. Participants provided emotional, psychological, and knowledge-based support to one another and emphasized the value of various intra-community supports for their well-being. This is clearly demonstrated when Bob stated:

*“Yeah, those were individuals who cared about me, and I cared about them; moreover, through the time when I gather these people, groups, A.A. [Alcoholics Anonymous], whatever group I’m in,… makes me blossom. …And I feel real good that I’ve got that support.”*

Here, the participant articulates not only the support they received from the community with which they engaged, but also how they provided support to the groups and gained mutuality and value from it. Additionally, they mention a connection with groups over extended periods and experiencing joy from being part of them.

*Self-Determined Care*

Participants expressed various levels of self-determined care in their quest to connect with healthcare. Here, self-determined care is the expression and importance of self-advocacy and fortitude to receive appropriate and relevant healthcare. This construct emerged with three subcategories: quality care, self-advocacy, and persistence.

*Quality Care*

Understandably, participants conveyed wanting to receive health care that met their needs. They were aware of the experiential differences between good and poor-quality care, and many of them were motivated to acquire good-quality care for themselves. Acquiring quality care as a marginalized individual requires fortitude and persistence. This was indicated by Alexis, a member of the Center Staff, when they said:

“I see what happens to older Black women, you know? (laughs) I’m just like ‘f***, this is not going to be easy.’ I’m watching it with my mom who just had a stroke two months ago. And so, I’m there [at the doctor] because I’m wanting to make sure that I take my care in my own hands. That I follow what I think are standard protocols around prevention. And then they are talking to me, and I’m feeling good, because I’m in a conversation with the doctor that I like.”

The above quote signifies great awareness of the health quality inconsistencies, the importance of health care, and perseverance. The participant is determined to keep advocating for quality healthcare until they are satisfied with what they receive.

*Self-Advocacy*

To receive health care that met their needs, participants expressed needing to advocate for themselves and be authentic with providers, including disclosing their LGBTQ+ identities. Beyond the awareness of the need to advocate for themselves in a society that is not keenly attuned to their needs, they also acknowledged learning the skills required to do so. Participants were acutely aware of being a minority population and therefore knew they must promptly disclose their identities to their health providers to meet their needs. Aaliyah, who identified as a Black transwoman, expressed this when she said:

*“Just like when I was at the emergency room the other last week. I had to let that doctor know, ‘Hey, I’m trans.’ She sitting here giving me a pregnancy test. I’m like, ‘Girl, I’m here for an abscess on my boob. I’m not here because I got knocked up and I’m pregnant or not.’ You know that could be fine for some trans people because they didn’t clock me, you know. But I’m coming here for real medical help. And I need you to look at me and make sure I’m okay. Not looking at me as a woman and that I’m pregnant. I can’t even get pregnant.”*

Here, the participant felt frustrated but determined to have her concerns addressed. She knew that she did not want resources wasted on unnecessary tests (pregnancy) and needed focused attention on the area of concern (abscess on breast). She was clear that she was not trying to “pass” and be seen as a cisgender woman and be treated as such. On the contrary, she was aware that her gender identity as a transwoman presented the need for distinct awareness required by the providers and was clear to point it out.

*Persistence*

Participants expressed having to be persistent and set boundaries to receive health care that met their needs. Persistence allows individuals to continue acting in the face of adversity and ultimately reach their desired outcome. Both perseverance and self-advocacy played an essential role in empowering study participants and helping them navigate complex systems. It enabled them to develop a voice and stand up for their rights. This was exemplified when Randy, an older adult, stated that

*“I was living in the mountains of [location], and I, at the time, was diagnosed with full-blown AIDS, and there was very little health care in the mountains. And we literally had to drive an hour to two hours down off the mountain to get health care and then drive back up the mountain. So, that facilitated my move back to [location] because, well, who doesn’t have better health care than [city’s name]? And I’m alive today to tell that story.”*

This participant is not only modeling advocacy skills for their peers in the room but also indicating a keen awareness of their medical needs. Knowledge of the self and the persistence in seeking quality care and advocacy enabled the participant to be clear and assertive in seeking the support they needed.

*Meaning-finding*

Although study participants expressed discriminatory and harsh environments in all sessions, they conceptualized an authentic life path moving towards health and wholeness. Participants voiced the desire to conceptualize their negative, hurtful experiences within the context of helping their intra-community members or the larger LGBTQ+ community. The act of meaning-finding was the focal category, encompassing all other identified categories. Two subcategories were identified within this construct: journey and progress over time.

*Journey*

Participants strongly reflected on their life path, journey, or story and its co-mingling with LGBTQ+ identities. Participants used their narratives to empower and teach other participants and make meaning of their lives. Participants also shared existential narratives in the sessions, harkening to “God” or a higher power as a reason for their existence. This was expressed when Jasmine, a TGGD woman, stated:

*“Too many things have happened to me in my life. Moreover, like I say, ‘Dear God, I know I’m here for an exact reason. There’s a reason why I’m here. Too many things have happened.’ And I believe that each one of us who are in the trans community needs to stand up and live our best life and be shiny little diamonds out there. And people will see us, love us, and respect us. Because what we have to do is put a little love in our hearts and let it flow through us. Let our love literally roar.”*

The participant was aware of the difficulties in life and reflected on her physical and temporal existence. She is also aware of new thresholds within her journey, along with the journey of her trans community. She invites them to express themselves and live fully, hoping that whole-hearted living would invite respect and love from the larger society.

*Progress Over Time*

Participants described their path to health and wholeness. They took retrospective glances at their journeys and identified how they had grown and matured over the years. This was indicated by Mark, a transman, who said:

*“But at a certain age… I think about how I was in my 20’s, I think about how I was in my 30’s. And now, at 50, it’s like all my problems, like you say, have to come from love. But see, I can’t love you, or [name] or [name] or, I can’t love none of them if I can’t love myself.”*

Here, the participant is looking at their past and distinguishing it from their present self. They are not disparaging their past selves but also identifying their new learning and growth. The participant is not only looking at themselves as an individual and what they can receive but also at how they can share a connection with others in the community.

## 5. Discussion

People need both self-connection (solitude) and community support (belonging) to move toward wholeness [75,80,81,82,85,86,87,88,89]. This study demonstrates how LGBTQ+ individuals developed this solitude and self-connection through understanding and affirming their intersectional identities, bolstering their authentic expression, and building upon their personal advocacy, demand for quality care, and persistence. Additionally, they built community support and belonging by navigating their identities within the LGBTQ+ community, by fostering a sense of community and belonging, and by building platforms for community support and advocacy. Meaning finding is intricately involved in each part of this process.

While this study draws on Minority Stress Theory, intersectionality, and existing models of wholeness, its primary contribution lies in advancing a process-oriented theoretical model that extends these frameworks rather than simply confirming them. Minority Stress Theory has been foundational in explaining how stigma-related stressors contribute to health disparities and how coping and social support can buffer their effects. This model builds on this foundation by specifying how individuals actively reorganize identity, meaning, and care engagement time.

In contrast to models that conceptualize meaning-finding, identity integration, and intra-community support primarily as coping or buffering mechanisms, the present findings suggest that these processes function as transformative mechanisms. Through these processes, individuals do not merely manage stress but actively reconstruct coherence, agency, and continuity in their relationship to healthcare. Healthcare encounters thus become sites where wholeness can be rebuilt or undermined, rather than only sources of stress exposure.

Conceptually, wholeness is distinguished from related constructs such as resilience, coping, or empowerment. Whereas resilience emphasizes adaptation under adversity and coping focuses on managing stressors, wholeness refers to the integration of identity, meaning, relational belonging, and self-determined engagement across life domains. Wholeness, therefore, captures a dynamic state of coherence that reshapes how healthcare is navigated and experienced, rather than a capacity to endure or respond to harm alone. Participants frequently described this coherence using construction metaphors—“building,” “brick by brick,” and “rediscovering who you are”—underscoring wholeness as an active integrative process rather than passive endurance.

A “divided life” is illustrated when individuals are unable to fully invest in their life work, remain in stunting relationships, develop harmful and negative feelings towards their peers, and hide or conceal their true identities for fear of being criticized and ostracized by their communities [75,85,86,87]. The Western medical model has struggled with an individual’s health. Modern medical knowledge, practices, and interventions have deep roots in extreme mind–body dualism [85,86,87], with little attention or value given to notions of holistic wellbeing and wholeness. However, it is recognized that an individual’s well-being is not simply based on interactions within their physical body but is a confluence of factors, including social determinants of health, community support, and psychological and spiritual health. Members of the LGBTQ+ community experience greater levels of oppression and stigma than the general population, often compounded by their intersectional identities [76]. MST has clearly articulated how these proximal and distal stressors increase LGBTQ+ health disparities [8,9,74], and previous research discusses coping strategies and long-term consequences on health for LGBTQ+ individuals [8,25]. Less work has been done on how LGBTQ+ individuals manage their inner wholeness by surmounting social barriers through self and community support. This study offers a model of LGBTQ+ inner wholeness while navigating the medical model.

Members of the LGBTQ+ community have repeatedly demonstrated resilience [14], grit, and perseverance in movements towards wholeness, recognizing the harms impacted upon them/us via structural, institutional, and interpersonal oppression and queer negativity [16]. These different forms of oppression are explicitly visible to community members as they navigate the medical model, seeking basic care for themselves and their community. This study reiterates previous literature [107], pointing to the series of destructive events LGBTQ+ individuals experience while pursuing healthcare. Participants navigated through multiple levels of structural and institutional inequalities, harmful medical practices and providers [108,109,110,111,112], and discriminatory insurance policies to seek primary and gender-affirming healthcare [109]. Participants also addressed negative messaging from the media and pathologizing by providers themselves, yet chose to pursue community building and resilience. These systemic barriers and non-comprehensive insurance policies often create financial barriers, which are further perpetuated by providers’ disrespect for community members. LGBTQ+-aware medical providers and researchers report that the burden of insurance policies and having to self-advocate often falls on LGBTQ+ individuals themselves [107]. Medical providers and staff were often ill-trained and lacked knowledge regarding LGBTQ+ health care issues or how to engage with them in meaningful and respectful ways. A review of TGGD inclusion in medical training identified a continued lack of training focused on patient–provider communication related to gender [113,114]. This lack creates further disrespect and disconnection of the LGBTQ+ community members from health care and promotes the deconstruction of their ties with health care [113,114].

However, several elements were identified that strengthen the ties LGBTQ+ individuals have to health care. Accessible and representative providers who can respectfully engage with LGBTQ+ individuals and provide them with appropriate health care play a critical part in supporting LGBTQ+ health. Additionally, care that affirms the individual’s identity, whether through gender identity affirmation, sexuality support, or holistic health, was critical. This includes avoidance of cisgender heteronormative assumptions and language, as well as openness towards multiple forms of being. LGBTQ+ individuals identified a need for and thrived on inter-community support. This was through organized support groups within the LGBTQ+ Center and, more holistically, through kinship (informal) families. Many TGGD members in the sample particularly noted intra-community support, identifying shared living spaces, clothing items, and improvements in gender-affirming care as salient factors. Another critical aspect of the reconstruction of connections to health care was community members’ capacity to separate health care from the ubiquitous Western medical model. Many members sought alternative methods of care and advocated for them across the community.

In a system of care that was not designed to serve the needs of the LGBTQ+ community, members find themselves struggling to navigate complex interactions while having their medical needs met in affirming ways. Participants in this study confirmed much of the past research on the interactions between LGBTQ+ individuals and medical providers [33,36,37,38,57,58,59,60,67]. Training for healthcare providers inclusive of LGBTQ+ health remains scarce [112], and knowledge may not be retained or sought after equally across providers. Providers with more than one marginalized identity (e.g., racial and sexual identities that are marginalized) they are more likely to score higher on LGBTQ+ cultural competency measures [113]. What arose from these sessions were elements larger than connection and disconnection to medical care.

The relationship between LGBTQ+ individuals and other LGBTQ+ community members is intricately bound, resulting from a long history of oppression [76]. Participants in this study strongly identified the need for connection with one another as critical to moving through the healthcare system. They relied upon recommendations for doctors, medications, treatment options, and specific medical practices to meet their needs. Their pain and traumatic experiences are echoed by peers and validated by collective empathy. Participants in each study group were distinct in specific ways, having different historical perspectives shaped by age and experience. However, the desire to be whole, to be seen, experienced, and appreciated as a full human being was primary. Whether individuals expressed this wholeness as a deep desire to reconnect disparate parts of their identities or to have a provider view them as more than one aspect of their identities (i.e., queer and Black and talented and outspoken), moving toward wholeness was omnipresent.

### Implications for Clinical Practice, Health Policy, and Medical Education

This study reframes healthcare encounters as cumulative experiences that can either reconstruct or fragment individuals’ sense of self and trust in care. Clinicians can promote reconstructive processes by engaging patients as epistemic partners, supporting identity integration, and recognizing how small, affirming, or dismissive actions accumulate over time. Such an approach emphasizes continuity, relational care, and attention to patients’ meaning-making processes. The findings highlight how policy environments shape the likelihood that healthcare systems function as reconstructing or deconstructing forces. Policies that mandate inclusive documentation systems, support sustained access to affirming care, and reduce reliance on patient self-advocacy may mitigate structural sources of harm. Integrating community-informed practices into policy design can further support equitable engagement in healthcare. These results suggest that preparing clinicians to provide affirming care requires more than discrete skills training. Medical education should incorporate longitudinal patient narratives, reflexive practice, and intersectional analysis to help trainees understand healthcare as a relational and life-course process. Embedding these perspectives may better prepare clinicians to support sustained connection and wholeness among LGBTQ+ patients.

*Limitations*

Several limitations warrant careful consideration when interpreting the findings of this study. First, this analysis represents a secondary use of qualitative data originally collected to develop causal loop diagrams examining connection and disconnection with healthcare. As such, the construct of wholeness emerged inductively through post hoc analysis rather than being the explicit focus of the original data collection. The absence of a structured model building guide in the parent study may have limited systematic exploration of some processes relevant to wholeness, and findings should be interpreted accordingly.

Second, the study sample consisted of individuals who were already connected to an LGBTQ+ community center, indicating that participants had, to some degree, overcome initial barriers related to isolation and access. This sampling context likely overrepresents individuals with greater access to intra-community resources and support, while underrepresenting those who are more isolated, disengaged from community spaces, or facing heightened structural vulnerability. As a result, the Constructing Wholeness model may be most applicable to community-connected LGBTQ+ populations and less explanatory for individuals without access to affirming social infrastructure.

Third, the sociopolitical and geographic context in which the study was conducted shapes the transferability of the findings. Access to community organizations, regional healthcare policies, and prevailing social attitudes toward LGBTQ+ identities vary widely across settings. Consequently, the pathways to wholeness identified here may operate differently—or be less accessible—in regions with fewer affirming resources, more restrictive healthcare systems, or heightened legal and social risks associated with disclosure.

It is also important to avoid idealizing intra-community support. While community connection emerged as a reconstructing force for many participants, such support is not uniformly available and may be uneven across subcommunities, geographic locations, or intersecting identities. In some contexts, intra-community spaces may reproduce exclusion, stigma, or resource scarcity, limiting their protective potential.

Taken together, these limitations suggest that the Constructing Wholeness model should be understood as a contextually grounded, process-oriented framework rather than a universal pathway applicable across all LGBTQ+ populations and healthcare systems. The model is most relevant to settings where some degree of community connection and access to care exists, and its applicability may be reduced in contexts characterized by extreme marginalization or structural exclusion. Future research should examine how wholeness is constructed under differing sociopolitical conditions, identify system-level leverage points that reduce deconstructing forces within healthcare, and explore how intersecting identities such as race, gender, age, disability, and nationality shape distinct pathways toward integration and coherence.

## 6. Conclusions

This secondary constructivist grounded theory analysis expands the original focus on connection and disconnection with healthcare by illuminating a broader process through which LGBTQ+ individuals move toward or away from wholeness as they navigate complex medical, behavioral health, and institutional contexts. Participants’ narratives demonstrated that experiences within healthcare settings do not simply accumulate as isolated encounters; rather, they shape and reshape one’s sense of self, agency, and belonging. The emergent theory of Constructing Wholeness in Connecting to Healthcare captures how individuals continually negotiate external reconstructing and deconstructing forces while, internally, engage in interconnecting selves, draw on intra-community support, practice self-determined care, and engage in meaning-finding. These processes together contribute to a dynamic and ongoing movement toward a more integrated sense of self.

Findings should be interpreted as reflecting perspectives of LGBTQ+ individuals and LGBTQ+-affiliated stakeholders embedded in community and care systems, rather than an exclusively service-user sample. Understanding these processes has critical implications for healthcare systems and professionals. Findings highlight the need for approaches that recognize LGBTQ+ individuals not merely as patients experiencing disparities, but as whole people navigating systems that can either fragment or support their well-being. Meaningful improvements in LGBTQ+ healthcare require not only enhanced provider knowledge and inclusive clinical practices but also structural changes that reduce stigma, expand access, and affirm the interconnected dimensions of identity, community, and embodiment. Supporting wholeness means attending to the relational, emotional, cultural, and structural aspects of healthcare, not solely the biomedical ones. Future research should explore how wholeness processes unfold across different sociopolitical environments, identities, and healthcare systems, as well as how community-based organizations can intentionally foster strengths-based and resilience-oriented models of care. Interventions grounded in the four wholeness processes identified here may hold promise for improving not only patient engagement and satisfaction but also long-term health trajectories. Ultimately, promoting wholeness in healthcare requires shifting from episodic, compliance-oriented models of care to relational systems that sustain identity safety, agency, and continuity across the life course.

## Figures and Tables

**Table 1:** Demographic Characteristics of Participants (*N* = 28).

Demographics	*N*	%
Gender identity		
Trans woman	6	21.4
Genderqueer/ Non Binary	4	14.3
Trans man	4	14.3
Cis woman	8	28.6
Cis man	4	14.2
Sexual orientation		
Gay/Lesbian	11	39.2
Queer	3	10.7
Heterosexual	6	25
Pansexual	3	10.7
Bisexual	2	7.15
Questioning	1	3.5
Race/Ethnicity		
White	16	59.3
Others	6	22.2

(1) % add up to greater than 100, because several participants chose multiple identities within each dimension. (2) Not all participants provided responses for each demographic variable; missing data are reported where applicable.

**Table 2:** Themes, Subthemes, and Illustrative Quotes.

Figure Domain	Construct	Illustrative Quote
External—Reconstructing	Accessible/Representative Providers	“They asked my pronouns right away and didn’t make it a big deal, which told me I wasn’t going to be judged.”
External—Reconstructing	Affirmative Care	“When I felt safe, I could actually focus on my health instead of worrying about being judged.”
External—Reconstructing	Inter-Community Support	“I didn’t know where to go until someone in the community told me, ‘This is the doctor you can trust.’”
External—Deconstructing	Structural Inequities/Discrimination	“It felt like I had to prove myself over and over just to get basic care.”
External—Deconstructing	Lack of Provider Knowledge/Training	“I was misgendered the entire appointment, and by the end, I just stopped correcting them.”
External—Deconstructing	Lacking Voice/Dismissed	“I kept trying to explain what I was experiencing, but it felt like they had already decided before I spoke.”
Internal—Inter-Connecting Selves	Re-Integrating Self	“Being able to bring my whole self into healthcare instead of splitting parts of myself off was huge for me.”
Internal—Self-Determined Care	Self-Advocacy	“I learned pretty quickly that I had to speak up for myself, because if I didn’t, things just wouldn’t happen.”
Internal—Meaning-Making	Journey	“Making meaning out of those experiences gave me a sense of control that the system had taken away.”
Outcome of Deconstructing Forces	Avoidance/Delayed Care	“I put off going even when I knew I needed care because I didn’t want to be hurt again.”

**Table 3:** Main Themes and Sub-Categories with Definitions.

Internal Process	Subcategory	Model-Consistent Definition
Inter-Connecting Selves	Re-Integrating Self	A process through which individuals reconnect fragmented aspects of self that have been disrupted by discrimination, stigma, or invalidation, restoring coherence between identity, embodiment, and self-worth.
	Intersecting Identities	Ongoing navigation and integration of multiple intersecting identities (e.g., gender, sexuality, race, age) as they shape healthcare experiences, vulnerability, and visibility.
	Authentic Expression	The capacity to express one’s identity openly and congruently in healthcare settings without concealment, justification, or fear of reprisal, enabling trust and relational safety.
Intra-Community Support	Navigating SGM Identities	Collective identity development through shared narratives, peer modeling, and exposure to diverse sexual and gender minority experiences within community spaces.
	(Intra-)Community Support/Advocacy	Emotional, informational, and instrumental support accessed through LGBTQ+ networks that facilitate healthcare navigation, buffer harm, and promote collective advocacy.
Self-Determined Care	Quality Care	The pursuit of healthcare that is responsive, affirming, and aligned with one’s lived needs and identities, informed by experiential and community-based knowledge.
	Self-Advocacy	Intentional efforts to assert needs, disclose relevant identities, and challenge provider assumptions to secure appropriate and respectful care.
	Persistence	Sustained engagement with healthcare systems despite prior harm or barriers, including boundary-setting, provider-switching, and continued care-seeking.
Meaning-Making (Integrative Process)	Journey	Reflexive interpretation of one’s healthcare and life experiences as part of a broader narrative of survival, purpose, and identity development.
	Progress Over Time	Recognition of growth, learning, and increased self-coherence across the life course, allowing past harm to be integrated into a developing sense of wholeness.

## Data Availability

The data supporting this study’s findings are available from the corresponding author upon reasonable request. The data are not publicly available due to privacy issues. Anonymized and de-identified transcripts may be provided if specifically requested.
